# Mixed messages: evaluating the concurrent presence of nutrition and health claims and front-of-pack warning symbols in five food categories in Canada

**DOI:** 10.1017/S136898002610189X

**Published:** 2026-02-02

**Authors:** Sarah-Maude Abran, Caroline Vaillancourt, Beatriz Franco-Arellano, Jennifer Lee, Sonia Pomerleau, Véronique Provencher, Marie-Ève Labonté, Lana Vanderlee

**Affiliations:** 1 Centre NUTRISS – Nutrition, santé et société, Institut sur la nutrition et les aliments fonctionnels (INAF), Université Laval, Québec, QC G1V 0A6, Canada; 2 École de nutrition, Faculté des sciences de l’agriculture et de l’alimentation, Université Laval, Québec, QC G1V 0A6, Canada; 3 Department of Nutritional Sciences, University of Toronto, Toronto, ON M5S 1A8, Canada; 4 School of Nutrition, Faculty of Community Service, Toronto Metropolitan University, Toronto, ON M5B 2K3, Canada

**Keywords:** Food labelling, Nutrition claims, Front-of-pack symbol, INFORMAS, Canada

## Abstract

**Objective:**

This study aimed (1) to characterise the use and prevalence of nutrition and health claims (NHC) and (2) to examine the association between NHC and the potential presence of Health Canada’s front-of-pack (FOP) nutrition symbol indicating high saturated fats, sugars and/or Na on a sample of Canadian prepackaged food products.

**Design:**

A cross-sectional analysis was conducted on five categories of prepackaged food products. Label components were classified using the International Network for Food and Obesity/non-communicable diseases Research, Monitoring and Action Support (INFORMAS) labelling taxonomy. Products’ nutritional profile was evaluated using Health Canada’s FOP symbol nutrient thresholds for saturated fats, sugars and Na.

**Setting:**

Data were obtained from the Food Quality Observatory database, collected between 2018 and 2022 from food retailers in Québec City and the Greater Montreal Area or online.

**Participants:**

A total of 2937 food products were evaluated from five food categories: breakfast cereals (*n* 392), cookies and granola bars (*n* 983), flavoured milks and plant-based alternative beverages (*n* 202), salty snacks and crackers (*n* 1063) and yogurts and plant-based yogurt alternatives (*n* 297).

**Results:**

Overall, 74·2 % of food products had an NHC and 28·9 % had an NHC and would require to display the FOP symbol. Food products that would require the FOP symbol were less likely to carry an NHC.

**Conclusions:**

The results demonstrate substantial use of marketing techniques highlighting positive product attributes. Given the potential for inconsistent messaging on food products carrying NHC and the FOP symbol, these results highlight an opportunity to improve Canadian labelling regulations by restricting the use of NHC on products high in saturated fats, sugars and/or Na.

Food labelling, including nutrient declarations, ingredients list, information on the presence of common allergens, health and nutrition-related claims and front-of-pack (FOP) labelling, is a fundamental component of food environments, serving to inform consumers and facilitate healthier dietary choices^(1)^. Food labelling is a commonly used policy approach, as it has broad reach and scope, providing information to consumers at the point of purchase and point of consumption. Nutrition information provided on the front of packages may have a particularly important influence on consumers, as this information is quickly available at the point of purchase and more likely to be viewed than information on the back of packages^(1)^. Key types of nutrition information on the front of packages are nutrition claims and FOP labelling systems, further defined below.

Claims are a common way to communicate information for consumers typically available on the front of packages. According to the *Codex Alimentarius* definition, claims are representations stating characteristics of a food product^(2)^. More precisely, nutrition and health claims (NHC) – such as ‘rich in fibre’ and ‘vitamin D improves Ca absorption’ – are regulated statements on food labels about products’ nutrient content or beneficial effects of product and/or its ingredients^(2)^. Food companies typically use NHC as part of labelling marketing strategies to inform consumers about characteristics of food products and emphasise the health attributes of products, thus positively influencing opinion of products and increasing the likelihood of purchase^(3)^.

NHC can increase the perceived healthfulness of food products by highlighting beneficial characteristics without disclosing unfavourable ones, creating a ‘health halo’ that may lead to misperceptions about the products’ overall nutritional quality^(4,5)^. Meta-analyses have shown that food products displaying NHC are more frequently chosen by consumers, regardless of the objective healthfulness of the product^(4,5)^. Although evidence suggests that consumers are better able to estimate a food product’s healthfulness when they consider nutrition claims in combination alongside nutrient declarations^(4)^, they tend to pay less attention to other nutritional information on the package when a nutrition claim is present^(6)^. There is inconsistent evidence as to whether food products that carry voluntary nutrition claims are healthier than foods that do not carry such claims^(7–10)^.

While the use of NHC in Canada is regulated as per the Food and Drug Regulations (FDR), their presence on food packages is voluntary^(11)^. Thresholds are provided for nutrition claims about beneficial nutrients such as fibres, vitamins and minerals for foods to be classified as ‘high in’ or ‘contains’ and for nutrients to limit such as Na, sugars or saturated fats for foods to be classified as ‘reduced’, ‘low in’ or ‘free’^(12)^.

FOP labelling systems have emerged internationally in an effort to make nutritional information more accessible and easy to understand, leading to better comparison between food products and supporting healthier choices^(13,14)^. Typically, FOP systems aim to reduce the consumption of food products high in nutrients associated with non-communicable diseases^(13)^. Canada introduced an FOP nutrition symbol in 2022 that was mandatory on products as of 1 January 2026^(15)^. The Canadian system includes a ‘high in’ warning symbol for prepackaged foods with excess saturated fats, sugars and/or Na^(15)^. According to Health Canada, these three nutrients were included as they were identified based on a review of the evidence to support consumer decision-making and decrease risk factors related to diet-related diseases^(16)^. Recommendations regarding these nutrients to limit are also found in Canada’s food guide^(17)^.

The presence of nutrition claims in the Canadian prepackaged food supply was last examined in 2013, whereby 46 % of products from twenty-three categories carried an NHC^(18)^. In addition, several studies have examined the expected prevalence of the FOP symbol on Canadian prepackaged food products when this label becomes mandatory, which is estimated to be approximately 60 % of prepackaged food products^(19,20)^.

In the current Canadian regulatory context, there is the potential for a product to simultaneously carry the FOP symbol and nutrition claims indicating positive characteristics of foods, which may result in consumer confusion^(21)^. Research has indicated that the presence of NHC on food products can mediate the effect of the FOP label on consumers as the health halo resulting from the claims remains, leading to an overall positive perception of food products despite the presence of a warning symbol^(22,23)^. Previous studies in Latin America examining the use of nutrition claims and FOP symbols have demonstrated a significant proportion of food products carried an NHC and simultaneously carried (or would carry) an FOP symbol^(3,24,25)^. To date, no studies have simultaneously examined nutrition claims and the potential presence of the FOP symbol as per the approved Canadian regulations on foods in the Canadian prepackaged food supply.

This study aimed (1) to characterise the use and prevalence of different types of NHC and the potential presence of the FOP nutrition symbol in a sample of Canadian prepackaged foods and (2) to examine the association between nutrition claims and the potential presence of the FOP nutrition symbol for products high in saturated fats, sugars and/or Na, overall and by food category. This study was conducted prior to the date of enforcement of the Canadian FOP nutrition symbol.

## Methods

Data were obtained from the Food Quality Observatory database^(26)^, which collects data on fifteen key food categories on an ongoing basis via collections for approximately three categories each year. Five broad food categories (incorporating seven sub-categories from the Observatory) of prepackaged foods were selected for this evaluation as they are known to often have high content in energy, saturated fats, sugars and/or Na^(20)^ and make a substantial contribution to Canadian diets^(20,27)^. These categories are also known to frequently carry marketing techniques on packages by food companies (e.g. using child-targeted marketing strategies)^(28)^. In addition, they represent recent data available from the Observatory prior to the official announcement of the forthcoming mandatory FOP regulations and thus can be considered baseline analyses prior to any changes that may have resulted from the FOP regulations.

Data for these categories were collected between 2018 and 2022 from food retailers from Québec City or the Greater Montreal Area, Canada. Briefly, data collectors visited the largest chains of supermarkets, big-box stores and speciality grocery stores or their online grocery stores. All food products corresponding with the specific food category were purchased by visiting retailers until saturation was reached. Research assistants then photographed all sides of each product, and photographs were coded for research projects as needed. Overall, 2937 products were evaluated from five food categories: (1) breakfast cereals (*n* 392, sampled in 2021); (2) cookies and granola bars (*n* 983, sampled in 2018/2019); (3) flavoured milks and plant-based alternative beverages (*n* 202, sampled in 2022); (4) salty snacks and crackers (*n* 1063, sampled in 2020); and (5) yogurts and plant-based yogurt alternatives (*n* 297, sampled in 2018/2019). Five products were excluded due to missing nutritional information on photos (*n* 3) or photos not being available (*n* 2). This study was conducted as part of the broader International Network for Food and Obesity/non-communicable diseases Research, Monitoring and Action Support (INFORMAS) Canada research programme^(29)^.

Nutrition information for products was double-coded to extract food and nutrition-related label information located anywhere on the package (product name, description, brand, UPC code, weight, ingredient list and Nutrition Facts table (NFt) values for thirteen available nutrients)^(20)^. Claims were identified and classified into nine categories using the health-related food labelling taxonomy developed by INFORMAS^(30)^ using an Excel spreadsheet. Table 1 describes the definitions of claims used in this study and the full list of labelling components that were extracted from product photos. Coding for claims was conducted by two independent trained research assistants, and double coding was conducted for a proportion of the products in each food category, at the beginning (10 % of each category) and at the end (> 2·5 % of each category) of the coding process. Disagreements resulting from the first round of double coding were reviewed with the senior researcher to achieve consensus, and claim definitions in the study codebook were adjusted for improved clarity. Double coding conducted at the beginning and at the end of the study suggested an agreement rate of 99·2 % and 99·8 % for claims coded, respectively. Using the extracted data, two additional categories of claims were established: claims related to the presence of nutrients for which public health recommendations would encourage greater consumption (fibre, vitamins and minerals, protein) called ‘high in nutrients to encourage’ claims and nutrients that public health recommendations suggest that consumers avoid or limit (sugars, Na, saturated fats, *trans* fats, total fats, cholesterol) called ‘low in nutrients to limit’ claims. Nutrients for these categories align broadly with Canada’s food guide^(31)^ and Canada’s Dietary Guidelines for Health Professionals and Policymakers^(17)^.

**Table 1.** Classification of claims and labelling components extracted, based on INFORMAS labelling taxonomy

Type of claim		Definition	Examples
Nutrition claims	Nutrient content claim	Information relative to the amount of a nutrient. Nutrients assessed: energy, cholesterol, saturated fat, *trans* fat, unsaturated fat, total fat, carbohydrates, sugar, Na, proteins, vitamins and minerals, fibres.	Source of protein 120 calories Unsweetened
	Nutrient comparative claim	Information relative to the amount of a nutrient in a food product, compared to the nutrient content in another product. Nutrients assessed: energy, cholesterol, saturated fat, *trans* fat, total fat, carbohydrates, sugar, Na, proteins, vitamins and minerals, fibres, non-nutritive sweeteners.	Reduced in Na Light More fibre
	Health-related ingredient claim	Information suggesting an ingredient is linked to health benefits or that is part of dietary guidelines. Ingredients assessed: wholegrain, fruits/nuts/honey, grains/seeds, vegetables/plants, probiotics/prebiotics, milk/cream, oils, cocoa.	Made with wholegrains Plant-based Cooked in 100 % avocado oil
Health claims	General health claim	Information relative to the beneficial effect of a food product, consumed in a healthy diet. Characteristics assessed: healthy mention, energy density, digestive health, bones health, oral health.	Healthy choice Ideal to keep you going between meals Energising
	Nutrient function claim	Information relative to the essential role of a nutrient or energy to maintain proper body functions and good health. Functions assessed: muscle, bone, growth, energy, strength, brain, nutrient absorption, digestion, immunity, overall health, other.	Protein helps build strong muscles Vitamin D improves Ca absorption Fe helps build red blood cells
	Disease reduction claim	Information relative to the reduced risk of developing a disease by eating a food product and its ingredients in a healthy diet. Diseases assessed: cardiovascular diseases, nutrient absorption, diabetes, osteoporosis, digestive health, cancer.	Oat fibre helps reduce/lower cholesterol, which is a risk factor for heart disease Contains Ca which reduces your risk of osteoporosis
Other claims	Quality and composition	Information relative to the composition of a food product or its quality, to stand out from similar products, implying or suggesting healthfulness.	All natural Canadian Vegan Contains no preservatives
	Intolerance and allergy	Information relative to the absence or potential presence of certain allergens, beyond requirements in the FDR^(11)^.	Lactose-free Manufactured in a facility that contains tree nuts, wheat gluten, dairy products, sesame, soya and sulphites
	Method of production and environment	Information relative to how the food product is made.	Kosher Organic Non-GMO Oven baked

INFORMAS, International Network for Food and Obesity/non-communicable diseases Research, Monitoring and Action Support; FDR, Food and Drug Regulations.

The nutritional profile of products was evaluated using Health Canada’s FOP nutrition symbol thresholds for saturated fats, sugars and Na^(15)^. The thresholds are based on the percent of daily values (DV) of the nutrients in the NFt. As shown in Table 2, the symbol is required when a food product exceeds ≥ 15 % of the DV for at least one of the three nutrients considered. For serving sizes ≤ 30 g or ml, the threshold is ≥ 10 % of the DV, and for packaged main dishes with a reference amount ≥ 200 g, the threshold is ≥ 30 % of the DV. The FOP labelling includes several exemptions described in the FDR^(11)^, including an exemption for sugar and saturated fats FOP symbol for dairy products (yogurts, cheese, kefir, buttermilk) if they contain ≥ 5 % DV of Ca and an exemption for milks for all three nutrients without consideration of the Ca content^(32)^. These exemptions are conditional, meaning that if the food product contains an added ingredient with saturated fats or sugar, the exemption for that nutrient is lost, thus triggering the FOP symbol according to the thresholds. Also, milk sold in glass containers is completely exempted from the FOP symbol^(32)^. These exemptions were considered in the current analysis for relevant products (yogurts and milks).

**Table 2.** Health Canada’s FOP nutrition symbol thresholds

Food product type	Saturated fats	Sugars	Sodium
Food products with a reference amount > 30 g or ml that are not main dishes	≥ 15 % DV		
Food products with a serving size ≤ 30 g or ml	≥ 10 % DV		
Packaged main dishes with a serving size ≥ 200 g	≥ 30 % DV		
Dairy products with ≥ 5 % DV of Ca	Conditionally exempted		≥ 15 % DV
Milk products in glass containers	Entirely exempted		

FOP, front-of-pack; DV, daily values.

Descriptive analyses characterised the frequency of various types of NHC and the proportion of the products that would carry the FOP nutrition symbol for at least one nutrient overall and by food category. Negative binomial regression models were used to examine differences in the number of NHC on products by food category. Logistic regression models were used to examine the difference in the prevalence of NHC on products by food category and the association between the presence of an NHC and the requirement for the FOP symbol, adjusting for food category. Sensitivity analyses tested a multiplicative interaction between the presence of NHC and food category, as well as the number of NHC and food category to see if these associations with the requirement for the FOP symbol differed between categories. When the interaction term was significant, associations were examined within categories. Analyses were conducted using SAS Studio (version 3.81, SAS Institute Inc.). A *P*-value < 0·05 was considered significant for all statistical analyses.

## Results

**Figure 1.**
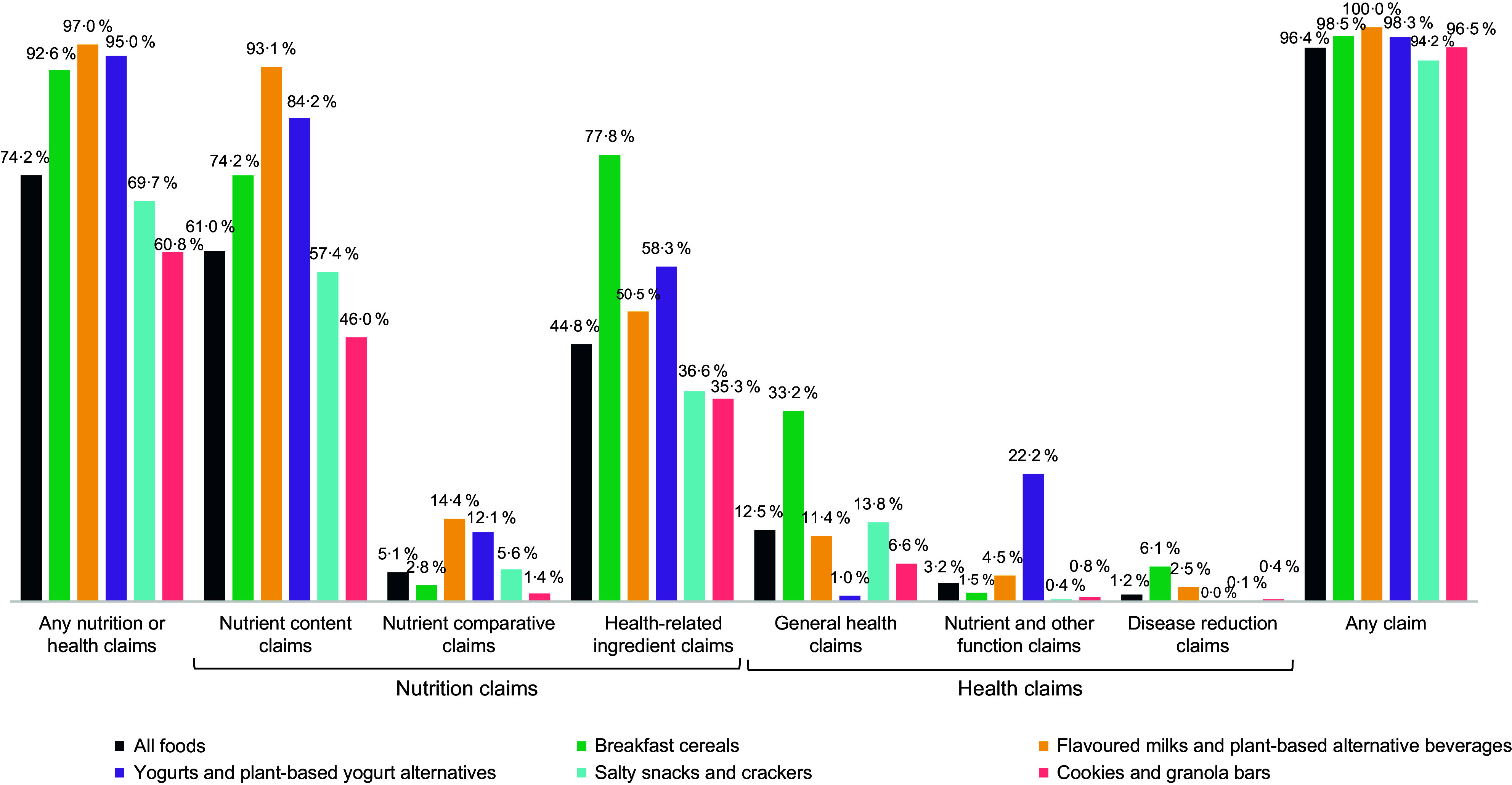
Frequency of all types of claims, overall and by food category.

Overall, 96·4 % (*n* 2937) of all analysed food products displayed at least one type of claim examined in this study (nutrition claims, health claims and other claims), indicating that almost all food products displayed a form of marketing (Figure 1). ‘Other’ claims were present on 92·6 % of all food products (see online supplementary material, Supplemental Figure 1 for results by food category). Among the entire sample, 15·0 % carried both at least one nutrition claim and one health claim.

### Presence of nutrition and health claims

NHC were present on 74·2 % of all products within the five categories; 73·4 % had a nutrition claim and 15·9 % had a health claim (see online supplementary material, Supplemental Table 1 for results by food category). NHC were present on more than 90 % of products in three categories (flavoured milks and plant-based alternative beverages; yogurts and plant-based yogurt alternatives; and breakfast cereals) (Figure 1). The prevalence of NHC differed by product category (see online supplementary material, Supplemental Table 2, for all comparisons). Flavoured milks and plant-based alternative beverages were more likely to have an NHC than breakfast cereals (OR = 2·61, 95 % CI 1·07, 6·39), salty snacks and crackers (OR = 14·20, 95 % CI 6·23, 32·32) and cookies and granola bars (OR = 21·03, 95 % CI 9·24, 47·87). Yogurts and plant-based yogurt alternatives were more likely to have an NHC than salty snacks and crackers (OR = 8·17, 95 % CI 4·78, 13·96) and cookies and granola bars (OR = 12·10, 95 % CI 7·09, 20·67), as were breakfast cereals (OR = 5·44, 95 % CI 3·65, 8·12 and OR = 8·06, 95 % CI 5·41, 12·01 compared to salty snacks and crackers and cookies and granola bars, respectively). Salty snacks and crackers were more likely to have an NHC than cookies and granola bars (OR = 1·48, 95 % CI 1·23, 1·78).

Nutrient claims (including nutrient content claims and nutrient comparative claims) were present on 62·1 % of all food products. Claims referring to energy were most frequent (21·6 %), followed by fibre (17·4 %) and *trans* fat (15·4 %) (Table 3). In addition to these nutrients, claims about non-nutritive sweeteners were present on 1·3 % of food products. Of nutrient claims, 32·7 % were ‘high in nutrients to encourage’ claims and 31·5 % were ‘low in nutrients to limit’ claims. Regarding nutrients included in Canada’s FOP labelling, 23·8 % of products had a ‘low in’ claim for saturated fats, sugars and/or Na.

**Table 3.** Frequency of nutrient claims for specific nutrients, total and by food category

		All foods (%)	Breakfast cereals (%)	Flavoured milks and plant-based alternative beverages (%)	Yogurts and plant-based yogurt alternatives (%)	Salty snacks and crackers (%)	Cookies and granola bars (%)
	Any nutrient claim	62·1	75·0	93·6	86·2	59·4	46·2
	Energy	21·6	18·1	38·1	9·8	20·9	24·0
Nutrients to limit	*Trans* fat	15·4	5·6	4·5	0·0	32·2	7·9
	Sugars	14·0	22·2	40·6	17·5	9·1	9·4
	Total fat	12·0	21·7	9·4	6·7	15·9	5·9
	Na	10·0	23·0	7·4	0·7	11·5	6·7
	Cholesterol	8·2	4·1	13·4	0·3	16·5	2·3
	Saturated fat	7·8	4·3	7·4	0·0	15·7	3·0
Nutrients to encourage	Fibre	17·4	55·6	4·5	2·7	10·9	16·3
	Vitamins and minerals	14·8	12·0	55·5	56·9	5·1	5·4
	Proteins	8·9	10·2	35·6	33·7	2·1	2·8

### Frequency of nutrition and health claims on food products by category

**Figure 2.**
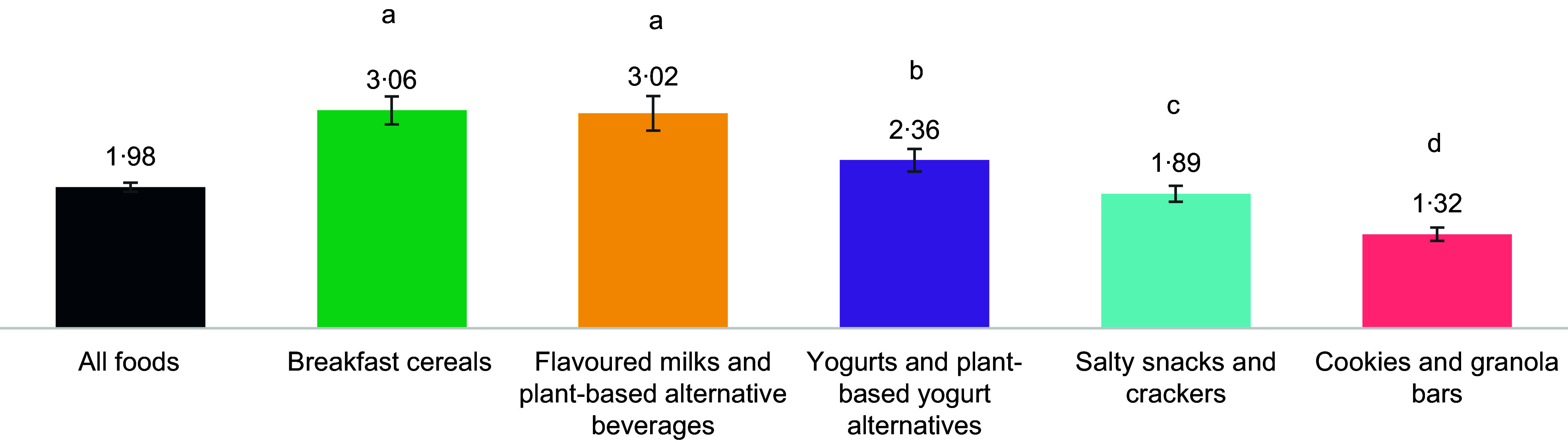
Mean number of nutrition and health claims, overall and by food category. Legend: different letters represent significant differences between categories (*P* < 0·05), as determined by a negative binomial regression.

Across the five categories, products had on average 1·98 NHC (sd: 1·83) (Figure 2). On average, breakfast cereals had more NHC than yogurts and plant-based yogurt alternatives (IRR = 1·29, 95 % CI 1·14, 1·46), salty snacks and crackers (IRR = 1·62, 95 % CI 1·47, 1·78) and cookies and granola bars (IRR = 2·32, 95 % CI 2·10, 2·57) (see online supplementary material, Supplemental Table 3 for all comparisons). Flavoured milks and plant-based alternative beverages had, on average, more NHC than yogurts and plant-based yogurt alternatives (IRR = 1·28, 95 % CI 1·10, 1·48), salty snacks and crackers (IRR = 1·60, 95 % CI 1·42, 1·81) and cookies and granola bars (IRR = 2·29, 95 % CI 2·02, 2·60). Yogurts and plant-based yogurt alternatives had, on average, more NHC than salty snacks and crackers (IRR = 1·25, 95 % CI 1·12, 1·40) and cookies and granola bars (IRR = 1·80, 95 % CI 1·60, 2·02). Salty snacks and crackers had, on average, more NHC than cookies and granola bars (IRR = 1·43, 95 % CI 1·32, 1·56).

### Products requiring the front-of-pack symbol

Almost half of all food products in the five categories examined (47·5 %) would require the FOP symbol for at least one nutrient as they exceed the thresholds for saturated fats, sugars and/or Na. The FOP symbol for saturated fats would be most frequent (25·8 % of products), followed by sugars (25·1 %) and Na (12·6 %). Cookies and granola bars had the highest proportion of products that would require the FOP symbol (69·7 %) (see online supplementary material, Supplemental Table 4). Cookies and granola bars were more likely to require the FOP symbol than breakfast cereals (OR = 5·15, 95 % CI 3·99, 6·64), flavoured milks and plant-based alternative beverages (OR = 4·33, 95 % CI 3·15, 5·97), yogurts and plant-based yogurt alternatives (OR = 6·03, 95 % CI 4·52, 8·04) and salty snacks and crackers (OR = 3·28, 95 % CI 2·73, 3·94) (see online supplementary material, Supplemental Table 2). Salty snacks and crackers were more likely to require the FOP symbol than breakfast cereals (OR = 1·57, 95 % CI 1·23, 2·01) and yogurts and plant-based yogurt alternatives (OR = 1·84, 95 % CI 1·39, 2·44).

### Nutrition and health claims and front-of-pack symbol

**Figure 3.**
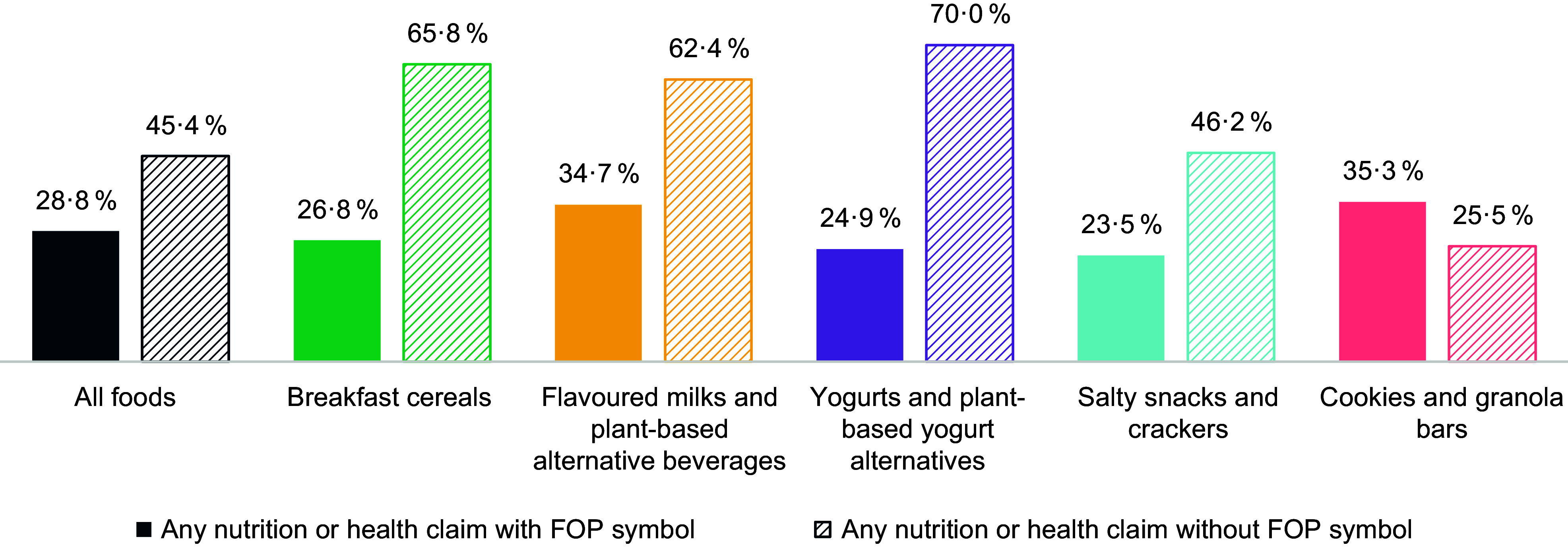
Frequency of products having any nutrition or health claims that would and would not require the FOP symbol. Legend: FOP, front-of-pack

Of all food products, 28·8 % had an NHC and would simultaneously require the FOP symbol (Figure 3). Among all food products, 18·7 % would require the FOP symbol and did not have an NHC, and 7·1 % did not require the FOP symbol and did not have an NHC. In an overall model adjusting for food category, food products with NHC were less likely to require an FOP symbol than food products without NHC (OR = 0·30, 95 % CI 0·24, 0·36), and the interaction between the presence of NHC and food category was NS (*X* ^2^ = 8·24, *P* = 0·08), suggesting that this was broadly consistent across food categories.

Flavoured milks and plant-based alternative beverages and cookies and granola bars had the most products that had NHC and would require the FOP symbol. Flavoured milks and plant-based alternative beverages were more likely to have an NHC and require the FOP symbol than breakfast cereals (OR = 1·45, 95 % CI 1·01, 2·09), yogurts and plant-based yogurt alternatives (OR = 1·60, 95 % CI 1·08, 2·36) and salty snacks and crackers (OR = 1·72, 95 % CI 1·25, 2·38), as were cookies and granola bars (OR = 1·49, 95 % CI 1·15, 1·93, OR = 1·64, 95 % CI 1·23, 2·21 and OR = 1·77, 95 % CI 1·46, 2·15, respectively) (see online supplementary material, Supplemental Table 2). Overall, simultaneous display of the FOP symbol and nutrition claims were more common than the FOP symbol and health claims (28·2 % *vs*. 6·4 %, respectively). See online supplementary material, Supplemental Table 5 for results within food categories. Among all food products studied, the FOP symbol for sugars would be most frequently required among food products with NHC (15·5 %; see online supplementary material, Supplemental Table 6). Supplemental Table 6 also shows results for saturated fats FOP symbol, Na FOP symbol and results within food categories.

Overall, 10·0 % of food products had ‘low in nutrients to limit’ claim (nutrient claim about sugars, Na, saturated fats, *trans* fats, total fats and cholesterol) and would also require the FOP symbol. Conversely, 10·4 % of food products had a ‘high in nutrients to encourage’ claim and would also require the FOP symbol. In the sample, 2·9 % of food products had either a saturated fats, sugars or Na claim and would require the FOP symbol for the corresponding nutrient.

### Number of nutrition and health claims

**Figure 4.**
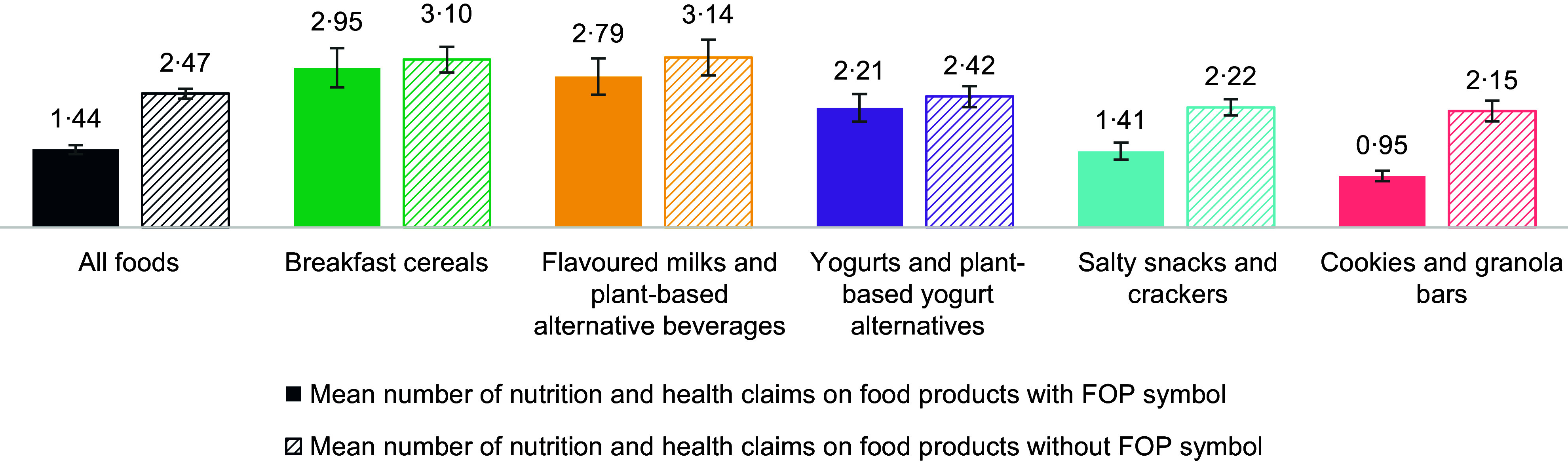
Mean number of nutrition and health claims on food products that would and would not require the FOP symbol. Legend: FOP, front-of-pack

Figure 4 shows the distribution of NHC on products that would require the FOP symbol overall and by category. On average, across all categories, there were fewer NHC on food products that would require the FOP symbol (IRR = 0·58, 95 % CI 0·54, 0·62). There was a significant interaction between the number of NHC and food category (*X* ^2^ = 71·94, *P* < 0·001), and results were thus stratified by category. There were on average fewer NHC on products that would require the FOP symbol in cookies and granola bars (IRR = 0·44, 95 % CI 0·39, 0·51) and salty snacks and crackers (IRR = 0·63, 95 % CI 0·56, 0·72), while there was no difference in the number of NHC on products that would and would not require the FOP symbol for the other three categories.

Breakfast cereals and flavoured milks and plant-based alternative beverages had the highest number of NHC with and without the FOP symbol. Breakfast cereals that would require the FOP symbol had, on average, more NHC than yogurts and plant-based yogurt alternatives (IRR = 1·34, 95 % CI 1·02, 1·75), salty snacks and crackers (IRR = 2·09, 95 % CI 1·72, 2·54) and cookies and granola bars (IRR = 3·10, 95 % CI 2·56, 3·74) (see online supplementary material, Supplemental Table 3). Flavoured milks and plant-based alternative beverages that would require the FOP symbol had, on average, more NHC than salty snacks and crackers (IRR = 1·97, 95 % CI 1·55, 2·51) and cookies and granola bars (IRR = 2·92, 95 % CI 2·31, 3·70), as did yogurts and plant-based alternatives that would require the FOP symbol (IRR = 1·56, 95 % CI 1·24, 1·98 and IRR = 2·32, 95 % CI 1·84, 2·92, respectively). Salty snacks and crackers that would require the FOP symbol had, on average, more NHC than cookies and granola bars (IRR = 1·48, 95 % CI 1·29, 1·70).

Breakfast cereals that would not require the FOP symbol had, on average, more NHC than yogurts and plant-based yogurt alternatives (IRR = 1·28, 95 % CI 1·13, 1·46), salty snacks and crackers (IRR = 1·40, 95 % CI 1·26, 1·55) and cookies and granola bars (IRR = 1·44, 95 % CI 1·28, 1·63), as did flavoured milks and plant-based alternative beverages that would not require the FOP symbol (IRR = 1·30, 95 % CI 1·11, 1·51, IRR = 1·42, 95 % CI 1·24, 1·62 and IRR = 1·46, 95 % CI 1·26, 1·69, respectively) (see online supplementary material, Supplemental Table 3).

## Discussion

### Summary of the study findings

The objective of this study was to characterise the use of NHC and assess the potential presence of Health Canada’s FOP symbol in a sample of Canadian prepackaged food products. NHC were found on almost three-quarters of the food products examined, most frequently on flavoured milks and plant-based alternative beverages, yogurts and plant-based yogurt alternatives and breakfast cereals, and nutrition claims were used more frequently than health claims. More than one-quarter of products that had an NHC would also require the FOP symbol. Food products that would require the FOP symbol were less likely to carry an NHC and had fewer NHC on average than food products that would not require the FOP symbol; however, the presence and number of NHC on foods that require the FOP symbol differed by category.

### Prevalence of use of claims

NHC were found on 74·2 % of products. The prevalence is higher among flavoured milks and plant-based beverages, yogurts and plant-based yogurt alternatives and breakfast cereals. Nutrition claims were more frequent than health claims. A previous analysis examining NHC on prepackaged foods with the INFORMAS labelling taxonomy in a larger sample of food products available for sale in Canada (including several overlapping categories with this study such as dairy products and substitutes, cereals and snacks in addition to other categories) found that 46 % of products had an NHC in 2010 and 43 % in 2013^(18)^. The lower prevalence of claims in these previous assessments compared to the present study may represent an increase in NHC use in the market, but may also be due to a different sample, which included more food categories, as well as differences in the classification of claims used, despite the use of a similar INFORMAS taxonomy. A study from Brazil examined a sample of food products with similar categories and found that 33 % of food products had an NHC^(3)^, which is much lower than the current study.

Analyses from other countries indicate some variations in the prevalence of claims and the number of claims on specific food categories. A study from New Zealand in 2014 found that 72 % of breakfast cereals had a nutrition claim, a somewhat lower prevalence than observed in the current study, and 40 % had a health claim, which is similar to the current results for this food category^(33)^. Among the few studies that have examined the mean number of claims on food products, two separate studies in New Zealand and Australia found the mean number of claims on breakfast cereals and yogurts and plant-based yogurt alternatives were, respectively, 4 and 5, somewhat higher than observed in the current study^(33,34)^. Again, classification of claims differed between the current and previous studies, which may in part explain these differences: the study in New Zealand used the standardised INFORMAS taxonomy, and the other study used the Australian government’s regulation classification for claims. These results may indicate regional regulatory differences in the use of NHC on food products and may also represent different strategies used by food companies in different regions to increase sales for various food categories.

Nutrition claims were present more frequently than health claims, similar to most other international studies^(3,8,9,18,24,25,34–36)^, but not all^(10)^. The use of health claims is much more strictly regulated than the use of nutrition claims in most countries, including Canada, with strict requirements on claim wording. These stricter regulatory requirements may make it more difficult for products to carry these claims and thus less likely to be used by the industry.

### Differences between categories

The current study found differences in the presence and frequency of claims between food categories, with claims more frequently present on flavoured milks and plant-based beverages and yogurts and plant-based yogurts alternatives. These results are consistent with a previous analysis of claims in Canada, which found the highest use of claims on dairy products^(18)^, higher than cereals and snacks. Similarly, studies in Latin America, Europe and Saudi Arabia concluded that dairy drinks or yogurts and breakfast cereals had a higher proportion of products with NHC than salty snacks^(9,24,25,36)^. This difference in NHC use between categories is perhaps unsurprising and is likely a result of product characteristics, as some categories include products with healthier nutritional profiles due to the nature and composition of those products, as well as broader marketing strategies, including child-targeted marketing, for some food categories such as breakfast cereals, which are more frequently marketed to children and perceived as healthy by consumers, as documented in other studies^(24,37,38)^. This may signal particular efforts by the food industry to highlight the positive attributes of these products.

### Simultaneous presence of the front-of-pack symbol and nutrition and health claims on product labels

Results showed that 28·8 % of food products had an NHC and would require the FOP symbol. The presence of NHC and FOP symbols is problematic from a consumer perspective, as conflicting information on products may lead to consumer confusion, and the health halo provided by NHC may obfuscate regulatory objectives to help consumers avoid products high in saturated fats, sugars and Na^(22,23,39)^. Similar studies in Latin America have shown that conflicting information from health claims and FOP labels is frequent^(3,8,24,25)^. For example, one study in Mexico conducted in 2017 prior to the implementation of an FOP policy found that 39 % of products would simultaneously carry an FOP symbol and some type of claim^(25)^, and a similar study in Brazil in 2019 suggested that 23·5 % of foods would simultaneously carry conflicting information^(8)^. There is also some evidence that there may be differences between nutrition claims and health claims: a study conducted in Brazil concluded that food products with nutrition claims were more likely to be high in critical nutrients, but food products with health claims were less likely to be high in these nutrients^(8)^.

Evidence has shown that effective food labelling regulations have led to reformulation of food products^(40,41)^. However, to date, few countries have regulated the use of nutrition claims on packaged foods in relation to FOP symbols to avoid conflicting messaging or information on products. In Argentina, a product required to carry an FOP symbol is not allowed to carry any complementary nutritional information and is prohibited from carrying complementary nutritional statements that highlight positive and/or nutritional qualities of the product^(42)^. In a somewhat similar fashion, Mexico and Colombia prohibit the use of health claims when a product has at least one symbol^(43,44)^. Regulations in Brazil, Mexico and Colombia are similar to those in Canada and only restrict nutrition claim use for ingredients indicated in the FOP symbol^(43–45)^. More coherent labelling regulations are likely to reduce consumer confusion, and restricting the use of NHC on packages that require FOP warning symbols is likely to encourage greater reformulation efforts by the industry.

The current study found that approximately 3 % of products would carry a claim for a nutrient for which it also exceeds the threshold and thus require the FOP symbol. The current study did not examine the content of these claims and whether or not they would be permitted to continue to carry these claims. While a minority of products was concerned, this potentially conflicting information may result in consumer confusion and may require additional consideration in forthcoming regulations.

### Nutritional quality of foods with nutrition and health claims

The current study found that products with NHC were less likely to be required to carry the FOP symbol and thus were less likely to be high in saturated fats, sugars or Na. Similar to the current study, a Peruvian study examining the presence of FOP symbols and nutrition claims after the implementation of an FOP ‘high in’ symbol policy in 2019 found that food products with an FOP symbol presented fewer health claims^(24)^. The majority of other studies have found that foods with NHC were less likely to be high in saturated fats, sugars and/or Na^(7,9,25,33,35)^. Some discrepant findings have been identified in Mongolian and Brazilian studies, which may be explained in part by less strict regulations for claims on food products, which allow the industry greater flexibility in using NHC as marketing tools, including on less healthy products^(10,46)^.

The classification systems to determine if foods are healthier or less healthy differed between studies, including whether nutrient profiling models included positive nutrients that are often associated with nutrition claims, which may influence the conclusions drawn from each study. Similarly, FOP thresholds vary between countries, which influences the prevalence of food products requiring an FOP and considered healthy or less healthy^(47)^. A previous Canadian study using the Food Standards Australia New Zealand Nutrient Profiling Scoring Criterion^(48)^ found that 42 % of their sample had an NHC and were considered ‘unhealthy’^(7)^, which is higher than the present study using the Canadian FOP symbol thresholds (28·8 %).

### Strengths and limitations

This study had several strengths. Double coding had a very high agreement rate, which indicates uniformity in claim classification. The standardised taxonomy used for the claim classification^(30)^ allows for the results to be compared to similar evaluations. The study also used the up-to-date Canadian regulations for the application of FOP symbols, including recently implemented major exemptions, to provide a portrait of the current policy situation. This study also had limitations. First, Health Canada released an interim update of the FOP symbol application for dense breakfast cereals in 2025 after analyses had been conducted^(49)^, which was not considered as it is subject to change in the official policy statement, but will likely affect the results related to the frequency of the potential presence of the FOP symbol for this food category. Another limitation is that only select categories were analysed, which does not provide a complete portrait of the use of NHC on all prepackaged foods in Canada. Given that the results differed by category, further research is needed to examine these associations in other food categories. The data from food packages were sourced from one province in Canada and may not be representative of all Canadian food supply, although it is unlikely that marketing or labelling techniques of major multinational food brands differ across provinces. In addition, data were collected between 2018 and 2022, and the use of NHC and the nutritional quality of foods may have changed during the period and since. In addition, as data collection was conducted over a period of five years, the differences found between categories may demonstrate the evolution of trends over time or changes in industry practices. As both secular trends driven by the food industry and policy-driven labelling changes continue to emerge, evaluations using more recent data are warranted to evaluate changes over time. Lastly, coding only captured one claim per nutrient (e.g. if there were multiple claims about vitamins and minerals on a package, only one was counted), and thus the estimates for the number of NHC are conservative and may underestimate the prevalence of use.

### Policy implications and future directions

NHC were abundant in the five food categories examined in the Canadian food supply. More than one-quarter of products in this sample would carry discrepant nutrition-related information (nutrition or health-related claims and symbols indicating high levels of nutrients) when the FOP symbol regulations become mandatory, which is likely to cause consumer confusion.

The current study did not assess where claims were made (on the front, side or back of packages) or their size and prominence, nor did it examine other marketing techniques that may be used to complement NHC such as marketing that appeals to children or corporate social responsibility campaigns. The current study also did not assess the number of nutrients indicated as ‘high in’ in the FOP symbol. In addition, there are important questions about how the use of NHC may change after the full implementation of the FOP symbol in Canada. Previous research has shown that health claims appeared on products more frequently after the implementation of a mandatory FOP symbol^(24)^. These results can be used in future research as baseline data to assess how industry use of NHC may change when the Canadian FOP regulations are fully implemented.

The results of this study highlight an opportunity to improve labelling regulations in Canada for food products having the FOP symbol by restricting the use of NHC when products are high in saturated fats, sugars and/or Na, as it has been suggested by Canadian experts^(50)^. Restricting mixed messaging about positive and negative attributes of food products could reduce consumer confusion and simplify nutrition labelling, thus helping consumers make more informed choices and further incentivising the food industry to reformulate their products to retain the right to use NHC on packages.

## Supporting information

Abran et al. supplementary material Abran et al. supplementary material

## Supplementary material

For supplementary material accompanying this paper visit https://doi.org/10.1017/S136898002610189X.
