# Supplementary material for: Mixed messages: evaluating the concurrent presence of nutrition and health claims and front-of-pack warning symbols in five food categories in Canada
Source: Public Health Nutr. 2026 Feb 2;29(1):e38. doi: 10.1017/S136898002610189X (PMC12964160; doi:10.1017/S136898002610189X)
Supplement: Abran et al. supplementary material [file S136898002610189Xsup001.docx]

**Supplemental material**

**Supplemental Figure 1. Frequency of food products having an other claim**

**Supplemental Table 1. Frequency of food products displaying a nutrition claim or a health claim**

| Food category | Nutrition claim (%) | Health claim (%) |
| --- | --- | --- |
| **All foods** | **73.4** | **15.9** |
| Breakfast cereals | 91.3 | 37.5 |
| Flavoured milks and plant-based alternative beverages | 96.5 | 16.3 |
| Yogurts and plant-based yogurt alternatives | 95.0 | 22.2 |
| Salty snacks and crackers | 68.9 | 13.9 |
| Cookies and granola bars | 59.8 | 7.3 |

**Supplemental Table 2. Difference between food categories for the prevalence of NHC and the FOP symbol requirement in logistic regression models**

| Food category comparisons | Prevalence of NHC | | FOP symbol requirement | | Prevalence of NHC and FOP symbol | |
| --- | --- | --- | --- | --- | --- | --- |
|  | OR | CI† | OR | CI† | OR | CI† |
| Flavoured milks and plant-based alternative beverages vs Yogurts and plant-based yogurt alternatives | 1.74 | 0.66-4.56 | 1.39 | 0.95-2.04 | 1.60* | 1.08-2.36 |
| Flavoured milks and plant-based alternative beverages vs Breakfast cereals | 2.61* | 1.07-6.39 | 1.19 | 0.83-1.70 | 1.45* | 1.01-2.09 |
| Flavoured milks and plant-based alternative beverages vs Salty snacks and crackers | 14.2* | 6.23-32-32 | 0.76 | 0.55-1.04 | 1.72* | 1.25-2.38 |
| Flavoured milks and plant-based alternative beverages vs Cookies and granola bars | 21.03* | 9.24-47.87 | 0.23* | 0.17-0.32 | 0.97 | 0.70-1.34 |
| Yogurts and plant-based yogurt alternatives vs Breakfast cereals | 1.50 | 0.79-2.86 | 0.85 | 0.61-1.19 | 0.91 | 0.64-1.28 |
| Yogurts and plant-based yogurt alternatives vs Salty snacks and crackers | 8.17* | 4.78-13.96 | 0.54* | 0.41-0.72 | 1.08 | 0.80-1.45 |
| Yogurts and plant-based yogurt alternatives vs Cookies and granola bars | 12.10* | 7.09-20.67 | 0.17* | 0.12-0.22 | 0.61* | 0.45-0.82 |
| Breakfast cereals vs Salty snacks and crackers | 5.44* | 3.65-8.12 | 0.64* | 0.50-0.82 | 1.19 | 0.91-1.55 |
| Breakfast cereals vs Cookies and granola bars | 8.06* | 5.41-12.01 | 0.19* | 0.15-0.25 | 0.67* | 0.52-0.87 |
| Salty snacks and crackers vs Cookies and granola bars | 1.48* | 1.23-1.78 | 0.30* | 0.25-0.37 | 0.56* | 0.46-0.68 |

†: 95% confidence interval

*p<0.05

**Supplemental Table 3. Difference between food categories for the frequency of NHC with and without FOP symbol**

| Food category comparisons | Frequency of NHC | | | |
| --- | --- | --- | --- | --- |
|  | With FOP | | Without FOP | |
|  | IRR | CI† | IRR | CI† |
| Flavoured milks and plant-based alternative beverages vs Yogurts and plant-based yogurt alternatives | 1.26 | 0.93-1.71 | 1.30* | 1.11-1.51 |
| Flavoured milks and plant-based alternative beverages vs Breakfast cereals | 0.94 | 0.72-1.24 | 1.01 | 0.88-1.17 |
| Flavoured milks and plant-based alternative beverages vs Salty snacks and crackers | 1.97* | 1.55-2.51 | 1.42* | 1.24-1.62 |
| Flavoured milks and plant-based alternative beverages vs Cookies and granola bars | 2.92* | 2.31-3.70 | 1.46* | 1.26-1.69 |
| Yogurts and plant-based yogurt alternatives vs Breakfast cereals | 0.75* | 0.57-0.98 | 0.78* | 0.69-0.89 |
| Yogurts and plant-based yogurt alternatives vs Salty snacks and crackers | 1.56* | 1.24-1.98 | 0.92 | 0.97-1.23 |
| Yogurts and plant-based yogurt alternatives vs Cookies and granola bars | 2.32* | 1.84-2.92 | 1.13 | 0.99-1.29 |
| Breakfast cereals vs Salty snacks and crackers | 2.09* | 1.72-2.54 | 1.40* | 1.26-1.55 |
| Breakfast cereals vs Cookies and granola bars | 3.10* | 2.56-3.74 | 1.44* | 1.28-1.63 |
| Salty snacks and crackers vs Cookies and granola bars | 1.48* | 1.29-1.70 | 1.03 | 0.93-1.15 |

†: 95% confidence interval

*p<0.05

**Supplemental Table 4. Frequency of food products having the FOP symbol for at least one nutrient and each nutrient individually**

| Food category | FOP symbol for at least one nutrient (%) | Saturated fats FOP symbol (%) | Sugars FOP symbol (%) | Sodium FOP symbol (%) |
| --- | --- | --- | --- | --- |
| **All foods** | **47.5** | **25.8** | **25.1** | **12.6** |
| Breakfast cereals | 30.9 | 13.5 | 19.9 | 3.1 |
| Flavoured milks and plant-based alternative beverages | 34.7 | 10.4 | 29.7 | 1.5 |
| Yogurts and plant-based yogurt alternatives | 27.6 | 7.1 | 22.9 | 0.3 |
| Salty snacks and crackers | 41.2 | 13.2 | 1.3 | 33.2 |
| Cookies and granola bars | 69.7 | 53.3 | 52.7 | 0.2 |

**Supplemental Table 5. Frequency of food products with a nutrition claim or a health claim that would require the FOP symbol**

| Food category | Nutrition claim and FOP symbol (%) | Health claim and FOP symbol (%) |
| --- | --- | --- |
| **All foods** | **28.2** | **6.4** |
| Breakfast cereals | 26.3 | 14.3 |
| Flavoured milks and plant-based alternative beverages | 34.2 | 7.9 |
| Yogurts and plant-based yogurt alternatives | 24.9 | 4.0 |
| Salty snacks and crackers | 23.0 | 5.6 |
| Cookies and granola bars | 34.5 | 4.7 |

**Supplemental Table 6. Proportion of food products having a NHC that would also require the FOP symbol**

| Food category | NHC and saturated fats FOP symbol (%) | NHC and sugars FOP symbol (%) | NHC and sodium FOP symbol (%) |
| --- | --- | --- | --- |
| **All foods** | **13.0** | **15.5** | **7.7** |
| Breakfast cereals | 13.3 | 16.3 | 2.3 |
| Flavoured milks and plant-based alternative beverages | 10.4 | 29.7 | 1.5 |
| Yogurts and plant-based yogurt alternatives | 7.1 | 20.2 | 0.3 |
| Salty snacks and crackers | 5.0 | 0.9 | 19.9 |
| Cookies and granola bars | 23.8 | 26.5 | 0.2 |
